# Physico-mechanical characterization and gamma radiation shielding capability of granodiorite from Suwayqat El-Arsha, Egypt; implications for construction and natural radiation shielding

**DOI:** 10.1038/s41598-025-33781-2

**Published:** 2026-01-17

**Authors:** Mokhles K. Azer, Ahmed M. Abdel-Rahman, Jason B. Price, Ahmed E. Abdel Gawad, Mohamed Y. Hanfi

**Affiliations:** 1 Department of Geological Sciences, National Research Centre, Cairo, 12622 Egypt; 2 Department of Geology, Faculty of Science, Al-Azhar University, Cairo, 11884 Egypt; 3 College of Earth, Ocean, and Atmospheric Sciences, Oregon State University, Corvallis, OR 97330 USA; 4 Ibex Exploration LLC, Lakewood, CO 80228 USA; 5 Nuclear Materials Authority, El-Maadi, P.O. Box 530, Cairo, Egypt; 6 Ural Federal University, Ekaterinburg, 620002 Russia; 7 Department of Physics, Dogus University, Dudullu-Ümraniye, 34775 Istanbul, Turkey

**Keywords:** Grandiorite, Mechanical characterization, Ornamental stones, Radiation shielding, Engineering, Environmental sciences, Materials science, Physics

## Abstract

The purpose of this study is to analyze the physico-mechanical characteristics of the Suwayqat El-Arsha area Granodiorite in the Eastern Desert of Egypt as a viable construction material and an effective natural radiation shield. The granodiorite was examined using twelve petrographic microscope thin-sections and twelve samples were analyzed via X-ray Fluorescence (XRF) for their chemical compositions. Both the physico-mechanical characteristics (density, water absorption, compressive strength, abrasion resistance, and flexural strength) were tested according to standard international test methods, ASTM. The gamma radiation shielding properties were evaluated over the energy range of 0.015-15 MeV using the Phy-X/PSD software program, which was based on the chemical composition and density values obtained from the XRF analysis. The analysis of these materials showed that they are mostly composed of silicon dioxide (SiO_2_ = 68.2–71.7 wt%), and their densities range from 2.61 to 2.73 g/cm^3^ with water absorption levels lower than 51%. The mechanical characteristics of these materials have been demonstrated through their mechanical properties such as compressive strength between 953 and 1146 kg/cm^2^, flexural strength between 15.2 and 27.5 MPa, abrasion resistance of 13.5–16.5 mm. As for attenuation coefficients; a decrease in linear attenuation could be seen from the beginning of 20.51 cm^− 1^ at 0.015 MeV until reaching 0.055 cm^− 1^ for 15 MeV. Subsequently, there has been an increase in half-value thickness from 0.034 cm to 12.4 cm. The effectiveness of these rocks in radiation protection was determined to be 100% at ≤ 0.03 MeV and ∼20% at 15 MeV. The Gd32 sample has the greatest density, mechanical properties, and shielding ability of all tested samples. The Suwayqat El-Arsha granodiorite has overall excellent structural durability and effective gamma-ray attenuation, making it an excellent alternative to traditional construction materials and radiation shielding materials, both because it is environmentally friendly and low-cost material.

## Introduction

As the use of ionizing radiation proliferates in several fields such as research, industry, medicine and disease management, and nuclear power generation, the need for safe protection from Radiological Hazards continues to escalate at an extraordinary rate. The introduction of gamma and X-ray technologies has allowed for the technological and medical advances that would not have otherwise been possible, but the uncontrolled release of these technologies has posed health risks and put the population at risk of genetic mutations and serious long-term consequences. Consequently, the development of effective, sustainable, and environmentally friendly radiation shielding materials is an essential component in establishing a protective barrier in all radiological environments^1–5^.

Traditionally, lead and lead-based materials have been the standard for radiation shielding due to their high density and high atomic number which provide good photon attenuation preservation.Nonetheless, in the advancement of greener/safe shielding, lead has its drawbacks with their high toxicity when non-radiating and their heavier weight which can become an issue when disposing of, as hazardous waste^5–10^. To mitigate these issues, scholars are working on non-hazardous shield materials which are environmentally friendly. Natural rocks and minerals have presented themselves as alternative potential shielding materials as they are abundantly available, low cost and have structural/chemical permanence. Recently, granitic rock, specifically granodiorite, have shown increased interest as prospected natural radiation shields due to their suitable composition and mechanical integrity^11,12^.

Granodiorite is composed mainly of quartz and plagioclase feldspar; whereas biotite and amphibole add to the rock’s distinct mineral composition and appearance.The presence of moderate concentrations of high atomic number elements such as, Fe, Ca, Ti, and Mg provides an improved potential for moderating ions. Granodiorite is physically and chemically stable, with sufficient density and strength to function as a material in nuclear facility construction, radiology laboratories, and places of radiation exposure. In addition, the aesthetics and physical characteristics of granodiorite provide dual functionality as a viable material for the shielding of ionizing radiation^13,14^.

In addition to natural materials such as granodiorite, advanced synthetic materials have also been investigated for radiation shielding purposes to meet modern safety and performance standards. These materials include heavy metal oxide glasses, polymer composites with high-Z nanoparticles (such as bismuth oxide, tungsten, and cerium oxide) as reinforcement, and lead-free ceramics designed to provide high attenuation efficiency and environmental safety^15,16^. Advanced materials can be developed at the micro- or nano-scale, in order to improve photon absorption, mechanical strength, and thermal stability while remaining either transparent or flexible, depending on the application. For example, borate- and tellurite-based glasses that are doped with heavy oxides demonstrate excellent gamma-ray attenuation properties that are comparable to lead-based materials but are non-toxic^17,18^. Developing advanced materials into hybrid composite systems with natural rocks such as granodiorite could enhance the overall performance of these materials, and provide additional value added benefits through natural cross-cutting surfaces. These advanced materials are promising candidates towards the development of multifunctional, sustainable, and efficacious radiation protection technology in the medical and industrial worldwide^19–28^.

In Egypt, granites are considered as one of the most resources bearing strategic and economic rare metals such as Zr, Nb, Ta, Be, B, Sn, W, F, Y, U, Th and REEs that take a remarkable interests of many researchers^29–31^. However, these granitic rocks are widely used as decorative ornamental stones, ancient heritage and modern constructions, which could be related to their high durability, compactness, fantastic colors and wide variety of appearances^32–36^. They are widely used as ornamental stones worldwide for a variety of purposes, including flooring, paving, cladding, funeral monuments, and statues. One of the main metrics used to assess the performance of ornamental stones is their durability, which can be improved by polishing techniques that lessen the stones’ capacity to absorb water vapor. Additionally, these procedures enhance the product’s visual qualities, like color and texture, which raises its market value^37,38^. As a result, natural stones have been utilized in construction extensively.

While separate investigations on granitic rocks have been performed in terms of their mechanical behavior or radioactivity, there is currently no complete investigation combining the petrographic, chemical, and mechanical integrity of a specific granodiorite type while also addressing the gamma ray attenuation characteristics. There is a significant gap in the literature with respect to the integrated assessment of granodiorite as both a structural and radiation-shielding material specifically from the Suwayqat El-Arsha area of the Eastern Desert. The Suwayqat El-Arsha granodiorite type has tremendous potential in Egypt’s construction industry due to its abundance and most importantly, it has not been adequately explored as a natural, lead-free eco-friendly radiation shield. There is an urgent need for a systematic study to confirm the dual purposes of Suwayqat El-Arsha granodiorite for structural applications and radiation protection. The main objective of the present work is to examine the primary petrophysical (porosity, dry bulk density, water absorption, and saturated-surface dryness) and mechanical (compressive strength, abrasion resistance, dynamic friction coefficient, and flexural strength) characteristics of Suwayqat El-Arsha granitoids as a case study of the Egyptian granites. Moreover, assessing granodiorite as a viable and sustainable gamma-ray shielding material, including the examination of its physical properties like density and water absorption, and its radiation attenuation properties such as the linear attenuation coefficients (LAC), half-value layers (HVL), mean free paths (MFP), and radiation protection efficiency (RPE) at a range of photon energies. Additionally, the research aims to relate these shielding properties to the chemical composition and mineralogy of granodiorite to help understand if this serves as a natural, environmentally friendly and lead-free shielding material in construction and nuclear facility applications.

## Geologic setting

**Fig. 1:**
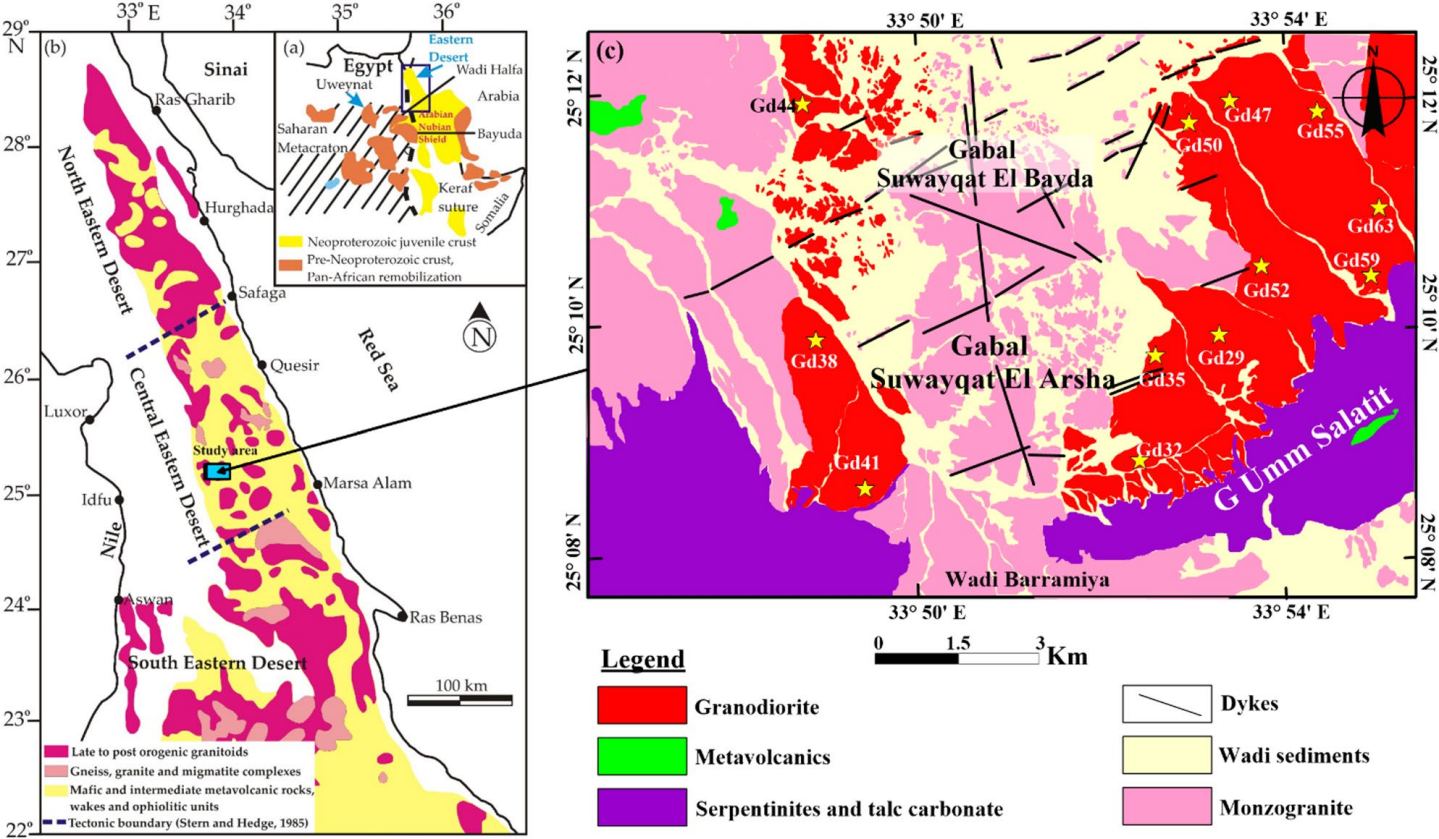
(a) Geologic map showing the Arabian Nubian Shield (ANS); (b) Geologic map showing Neoproterozoic basement rocks in the Eastern Desert (ED), Egypt^39^; (c) Geologic map of Suwayqat El-Arsha area.

The investigated Suwayqat El-Arsha area is exposed in the Egyptian Eastern Desert as an interested part of the Arabian–Nubian Shield (ANS) (Fig. 1a, b)^39^. The investigated area comprises serpentinite and related talc carbonate, metavolcanics, granodiorite and monzogranite that were dissected by trachyte and dolerite dykes and quartz veins (Fig. 1c).

**Fig. 2:**
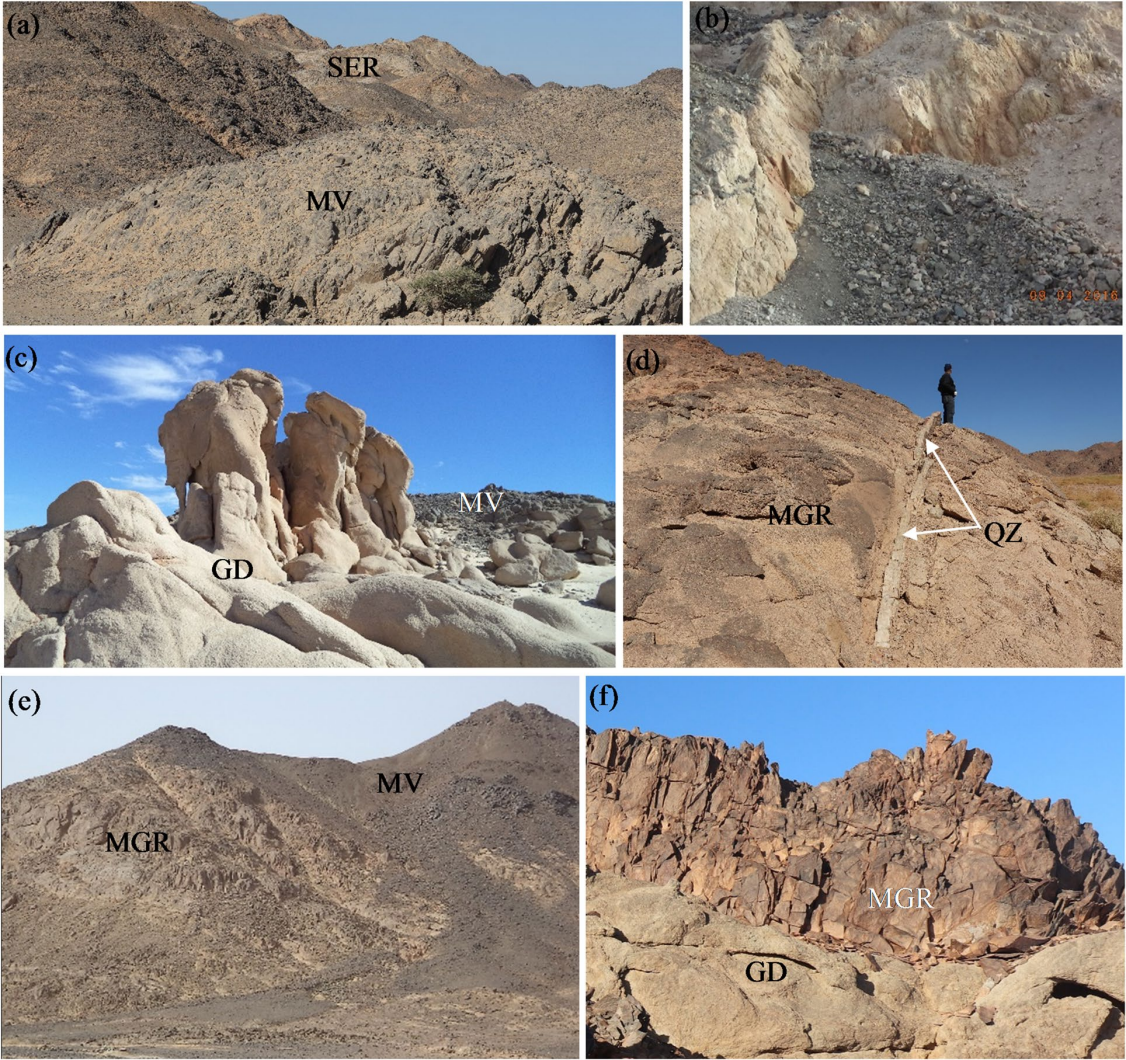
(a) Serpentinite rocks were thrusted over the metavolcanics along NE trend, (b) Close up view of pale gray talc carbonates, (c) Highly weathered granodiorite showing monumental shapes intruded the metavolcanics, (d) Quartz vein invaded the monzogranite along NE trend, (e) Sharp intrusive contact between the monzogranite and the metavolcanics, (f) Monzogranite intrudes the granodiorite.

The most important serpentinite masses are well represented in the Umm Salatit and west Suwayqat El-Arsha areas (Fig. 1c). These rocks show moderate relief, fine-grained, varied from dark grey to violet colors, highly deformed and foliated particularly along their peripheries. The talc carbonate rocks are mostly encountered with the serpentinite rocks, and is distinguished by softness, buff creamy colors and cavernous appearances (Fig. 2a, b). Chromite lenses with limited extension are well observed, and mostly encountered with some serpentinite rocks along NE trend. These rocks were thrusted over the metavolcanics (Fig. 2a), and were intruded by granitic rocks with sharp intrusive contacts.

The metavolcanics are well recorded in the southeastern and northwestern parts of the investigated area. They comprising a thick sequences of stratified lava flows comprising metabasalt, meta-andesite, metadacite and metarhyolite, which are intercalated with their pyroclastics. These rocks exhibit highly foliation, altered and varied from black, gray, greenish-gray, buff to reddish-brown colors. They are well distinguished by fine-grained textures, well-defined layering and remarkable hydrothermal alteration. The pyroclastics are consisted of ash, lithic and lapilli tuffs that were have been derived from the same composition of their lava.

Older granitoids are predominated in the investigated area, are distinguished by low- to-medium relief, medium- to coarse-grained, whitish gray to gray colors. They are highly fractured, jointed, weathered, altered, exfoliated and showing monumental shapes and bouldery appearance (Fig. 2c). They are dissected by N–S strike slip fault with sinstral lateral displacement. Pegmatite pockets, dykes and quartz vein invaded the older granitoids.

Muscovite monzogranite is widely distributed plutons, leucocratic, pink to reddish pink colors and distinguished by weathered surface (Fig. 2d). It occurs as medium- to coarse grained, moderate- to high relief, and intrudes the older granitoids and the surrounding serpentinite rocks with sharp intrusive contacts (Fig. 2e, f). This rock is highly jointed, fractured, cavernous landforms and exfoliated along E–W and N–S trends. The monzogranite contains xenoliths of mafic rocks and older granitoids.

Dyke swarms and quartz veins are widely distributed in the basement rocks in the prospected region. Trachyte dykes are massive, fine-grained, reddish brown to pale brown and buff colors, and have NNE and N–S trends. They are usually the most predominated one, varied from 5 to 10 m thick, and stained by iron oxides. These dykes are dissected the investigated granites in the prospected area. Dolerite dykes are fine-to medium-grained, massive, greenish gray to green colors and range from 2 to 4 m thick. They have NNW and N–S trends, and predominated in the investigated granitic rocks. Quartz veins are distinguished by milky white and reddish brown due to the effect of hematitization processes. They are varied from 0.5 to 3 m thick, have E–W, NW and NE, and are widely distributed in granitic rocks (Fig. 2d).

## Materials and methods

### Field and analytical investigations

The field and laboratory investigations conducted for this project are summed up as follows:

The field investigation involved sampling and describing the field relationships and structural settings of exposed rock units, particularly granitic rocks, which are the main target of the present study. The petrographical study of twelve thin sections for the investigated older granitoids in the prospected area were carried out with a polarized light microscope at the National Research Center in Dokki, Egypt. The collection of twelve granodiorite specimens was based both on field observations and consistency from a petrographic standpoint, which demonstrate little to no lithological variability of the Suwayqat el-Arsha granodiorite, as a result; these twelve specimens meet the sample requirements set down by other studies involving the mechanical/radiological properties of natural stone. Most studies consider 8 to 15 samples sufficient to represent a specific type of rock. In measuring all physical/mechanical/density-related parameters, three measurements of each variable were carried out in order to reduce the margin of error from the measurement process and increase the statistical confidence level of the values obtained. The current study’s objective is to characterize a homogeneous lithological unit; therefore, the number of samples used was statistically adequate to produce representative values for petrography or the mechanical and gamma attenuation properties associated with the original lithology. Chemical analysis of twelve samples were have been analyzed at the Northwest University, China in order to determinet the major elemental oxides chemical composition of the granodiorite and presented in Table 1. Powdered samples were heated at 1000 °C for 50 min to determine Loss on Ignition (L.O.I.). Samples were prepared for X-ray fluorescence (XRF, Rigaku RIX2100) by mixing 0.7 g of sample with 0.3 g of LiF, 0.4 g of NH_4_NO_3_, 3.6 g of Li_2_B_4_O_7_, and 2–3 drops of 1.5% (w/w) LiBr solution. This mixture was melted in a non-wetting precious metal crucible and poured into a glass disk prior to analysis. The analytical analyzed data have been done utilizing international rock standards for calibration such BCR-2, BHVO-1, and AGV-1^40^. The analytical precision of this protocol, based on sample replicates, is better than 1% for major oxides.

**Table 1:** Chemical analyses of major oxides (wt, %) for granodiorite.

Sample No.	Gd29	Gd32	Gd35	Gd38	Gd41	Gd44	Gd47	Gd50	Gd52	Gd55	Gd59	Gd63
SiO_2_	68.21	68.84	69.82	70.16	70.65	68.58	71.69	71.37	70.26	71.44	71.32	69.42
TiO_2_	0.77	0.67	0.51	0.46	0.45	0.74	0.26	0.34	0.54	0.24	0.42	0.59
Al_2_O_3_	14.88	14.38	14.73	14.9	14.7	14.61	14.71	14.56	14.81	14.92	14.49	14.29
Fe_2_O_3_	3.32	2.92	2.68	2.48	2.44	3.26	1.99	2.31	2.6	2.12	2.38	2.89
MnO	0.07	0.06	0.07	0.07	0.07	0.07	0.07	0.06	0.07	0.07	0.06	0.07
MgO	1.56	1.23	1.03	0.89	0.88	1.53	0.92	0.89	1.06	0.95	0.91	1.25
CaO	3.87	3.33	2.89	2.77	2.59	3.73	2.37	2.34	2.97	2.42	2.41	3.07
Na_2_O	3.56	3.63	3.67	3.84	3.63	3.56	4.09	3.66	3.63	3.95	3.75	3.54
K_2_O	2.54	2.92	3.08	2.9	3.19	2.72	3.44	3.57	3.07	3.23	3.55	3.19
P_2_O_5_	0.14	0.11	0.1	0.09	0.09	0.13	0.08	0.08	0.09	0.06	0.08	0.11
LOI	0.85	0.69	0.7	0.59	0.56	0.82	0.5	0.55	0.6	0.54	0.57	0.64
Total	99.76	98.78	99.28	99.15	99.25	99.75	100.12	99.76	99.7	99.9	99.94	99.06
Density±0.001 (g/cm^3^)	2.61	2.73	2.69	2.70	2.66	2.63	2.69	2.71	2.63	2.66	2.67	2.62

### Physico-mechanical properties

**Fig. 3:**
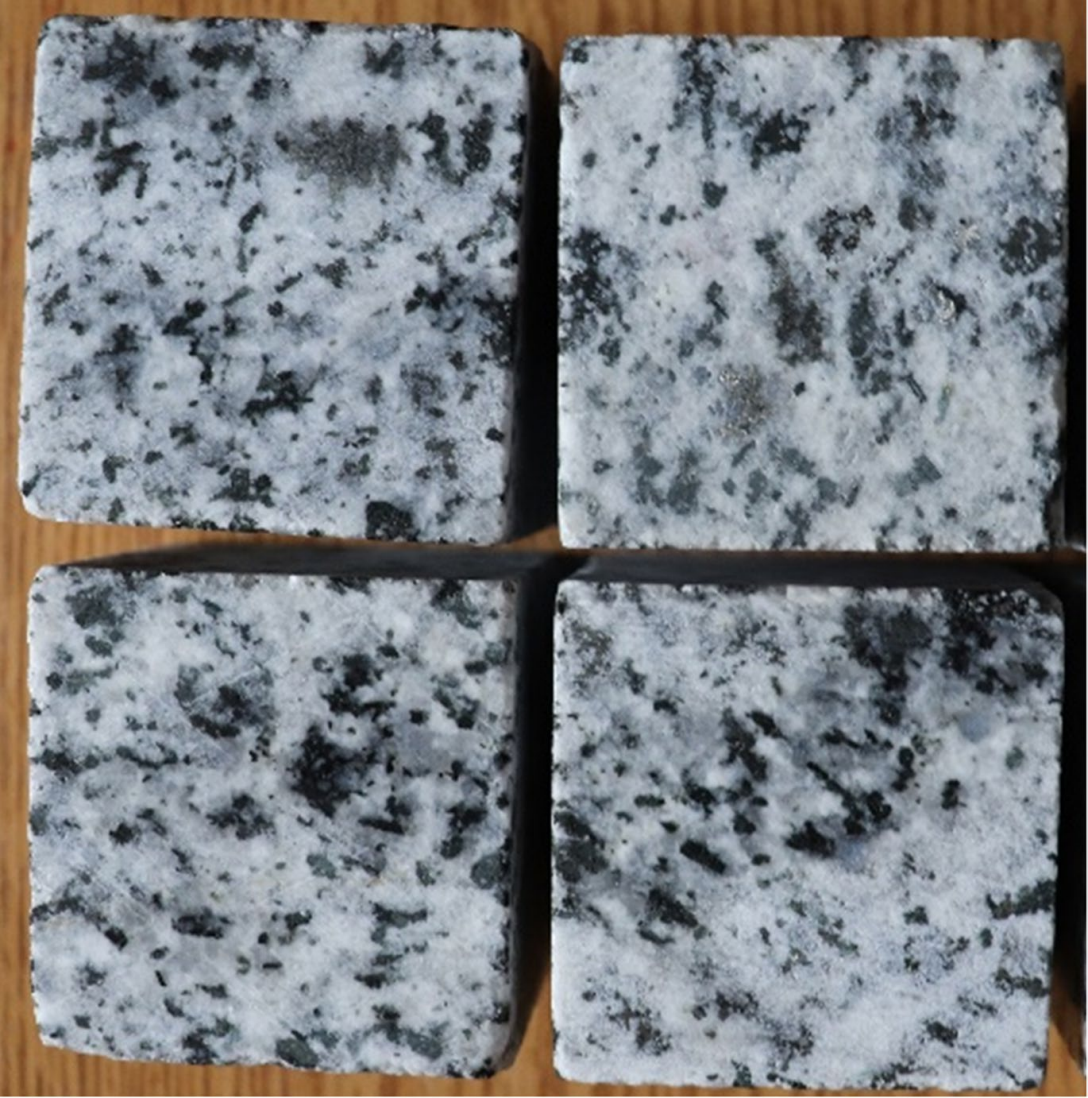
Photographs showing the examined white to black and gray granodiorite at Suwayqat El-Arsha, Central Eastern of Egypt.

Petrophysical-mechanical characterization of dimension stone is crucial for determining its mechanical behavior and assessing its durability. The measured physical properties, including apparent porosity, dry bulk density, water absorption, and saturated-surface dryness (SSD), which were assessed for 12 samples of granodiorite from which approximately cubic specimens (4 × 4 × 4 cm) were cut (Fig. 3).

Granodiorite samples used in this research were taken from the chosen area and subsequently prepared for density measurements. Each sample was cleaned, cut into comparable cubic shapes, and polished to remove surface impurities and irregularities. The samples were placed in an oven for 24 h at 110 °C to remove all remaining moisture that may interfere with mass measurement.

In addition to physical properties, the mechanical properties were measured, including compressive strength, abrasion resistance, and flexural strength. These properties are important because they measure the loading capacity of the rocks of interest (Table 2), which is one of the most crucial pieces of information in many engineering domains, including mining, tunneling, and road design^41^. The standardized mechanical tests were conducted in the Housing and Building National Research Center (HBRC) at the Laboratory of Raw Building Materials and Processing Technology Research Institute, Dokki, Egypt, with standard test equipment to develop reproducible mechanical testing results. To measure the compressive strength of the materials tested, a Controls Testing Machine (Model 50-9020 with a 2000kN capacity) was utilized as per ASTM C170^42^ and the compressive strength test was conducted at 0.5 ± 0.05 MPa/s. Flexural strength was determined with a Controls Universal Testing Frame (Model 65-L26) under a three-point bending configuration and at a crosshead speed of 1.0 mm/min - as per ASTM C880^43^. The abrasion resistance of the materials tested was assessed with a Böhme Abrasion Tester (DIN 52108) using a standard load of 294 N and a speed of 30 ± 1 rpm^44^. Minimization of uncertainties during sample preparation was achieved by taking samples with dimensions of 4 cm (length) × 4 cm (width) × 4 cm (height); dimensional tolerances were set at ± 0.3 mm, flatness tolerances were set at ± 0.1 mm, and residual moisture was maintained through drying the samples in the oven at 110 °C for 24 h. The minor uncertainties associated with micro-fissures caused by sawing and the lack of homogeneity of mineral distributions were reduced by testing multiple samples (*n* = 3–5) for each property being tested.

**Table 2:** Mechanical properties of granodiorite samples used in the study.

Sample No.	Compressive strength (kg/cm^2^)	Abrasion resistance (mm)	Bending resistance (MPa)	Water absorption (%)
Gd29	1047	15.5	15.2	0.51
Gd32	1146	15	27.5	0.21
Gd35	1102	16.5	20.1	0.38
Gd38	1016	13.5	19.6	—
Gd41	953	16.5	23.2	—
Mean	1053	15.4	21.12	0.367
SD	75	1.24	4.57	0.15

After drying the samples, they were placed in the desiccator until they cooled to room temperature for weighing. To measure the density (ρ) of the granodiorite samples, Archimedes’ principle was used. Archimedes’ principle utilizes the property of buoyancy, which is the force that is exerted on an object submerged in a fluid. The measurements were taken with a specific density determination kit connected to a high-precision analytical balance. Each sample has its weight in the air (W_a_) measured as well as weight in distilled water (W_l_). The density is subsequently calculated with the following relation^45^:

1 $$\rho =\frac{{{w_a}}}{{{w_a} - {w_l}}} \times {\rho _l}~$$

where ρ_l_ is the density of the liquid (distilled water at 25 °C is 0.997 g/cm^3^). For each run, three measurements were taken and the average was determined to yield the density of the sample. This provides a reliable way to measure bulk density which is an important factor affecting the attenuation of properties of the samples to gamma radiation.

Subsequently, the experimental measurement of the water absorption capacity for the granodiorite samples was conducted in accordance with Eq. (2).

2 $${\mathrm{Water~absorption~}}\left( {{\mathrm{K}},{{\% }}} \right)=~\frac{{\left( {{W_{sat}} - {W_{dry}}} \right)}}{{{W_{dry}}}}~ \times 100$$

where, W_sat_ is the weight of sample at saturated water and W_dry_ is the weight of sample in the dry air.

To evaluate and determine the gamma-ray shielding properties of granodiorite samples, baseline shielding parameters were estimated with the use of the Phy-X/PSD software^46^. The Phy-X/PSD software is a web-based open-access computational tool and serves as a widely used mechanism for assessing photon interaction parameters and radiation attenuation properties of materials. The software estimates a range of photon attenuation parameters across a wide range of photon energies (0.015–15 MeV) requiring only the density and chemical composition of material input data. The parameters provided insightful data regarding the interaction response of incident photons to the granodiorite matrix.

The mass attenuation coefficient (MAC) was calculated using the weight fractions of elements that constitute each granodiorite sample. The MAC is defined as the probability of photons interacting with one unit of mass, and is fundamental to the attenuation of gamma radiation. The MAC of each granodiorite was determined from the equations^47,48^:

3 $${\text{MAC }}\left( {{\mathrm{c}}{{\mathrm{m}}^{\mathrm{2}}}/{\mathrm{g}}} \right){\text{ }}=\sum {{\mathrm{w}}_{\mathrm{i}}}{\mathrm{~}}\left( {{\mathrm{LAC}}/{{\mathrm{\boldsymbol{\uprho}}}_{\mathrm{i}}}} \right)$$

where *w*_*i*_ is the weight fraction and (LAC/ρ)_i_ is the mass attenuation coefficient of the ith element.

The linear attenuation coefficient (LAC) describes the proportion of incident photons (I_o_) that are either transmitted (I) per unit thickness (x, cm) of the material. The LAC can be calculated from the MAC values using the presented aspects^47,58^:

4 $${\text{LAC }}\left( {{\mathrm{c}}{{\mathrm{m}}^{ - {\mathrm{1}}}}} \right){\text{ }}=\frac{1}{X}\ln \frac{{{I_o}}}{I}$$

The half-value layer (HVL) refers to the thickness of the material needed to attenuate the intensity of the incident photons to 50% of the original value. It is a useful metric for measuring shielding effectiveness and is determined byequation^47,49^:

5 $${\text{HVL }}\left( {{\mathrm{cm}}} \right)\,=\,{\text{ln 2}}/{\mathrm{LAC}}$$

A lower HVL value indicates a more efficient shielding material.

The mean free path (MFP) is the average distance a photon travels in the material before an event occurs (absorption or scattering). It calculated as equation^49^:

6 $${\mathrm{MFP~}}\left( {{\mathrm{cm}}} \right)={\mathrm{~}}\frac{1}{{{\mathrm{LAC}}}}$$

A smaller MFP demonstrates a higher likelihood of interacting with photons in the granodiorite structure, substantiating its use as an efficient radiation shielding material.

## Results and discussion

### Petrography of granodiorite

**Fig. 4:**
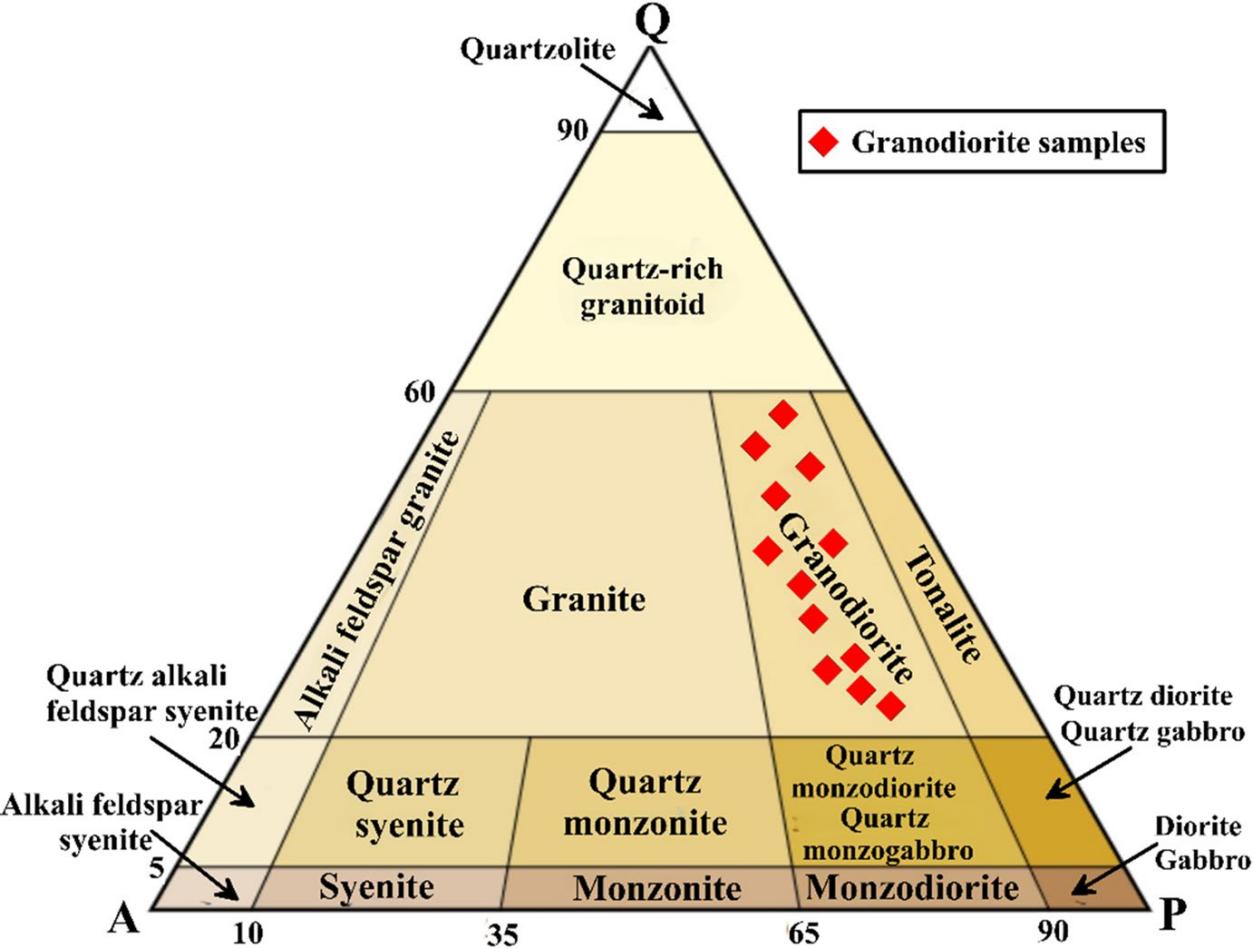
The Q-A-P ternary diagram after^50^ showing the modal analyses plots for the investigated granitoids.

**Fig. 5:**
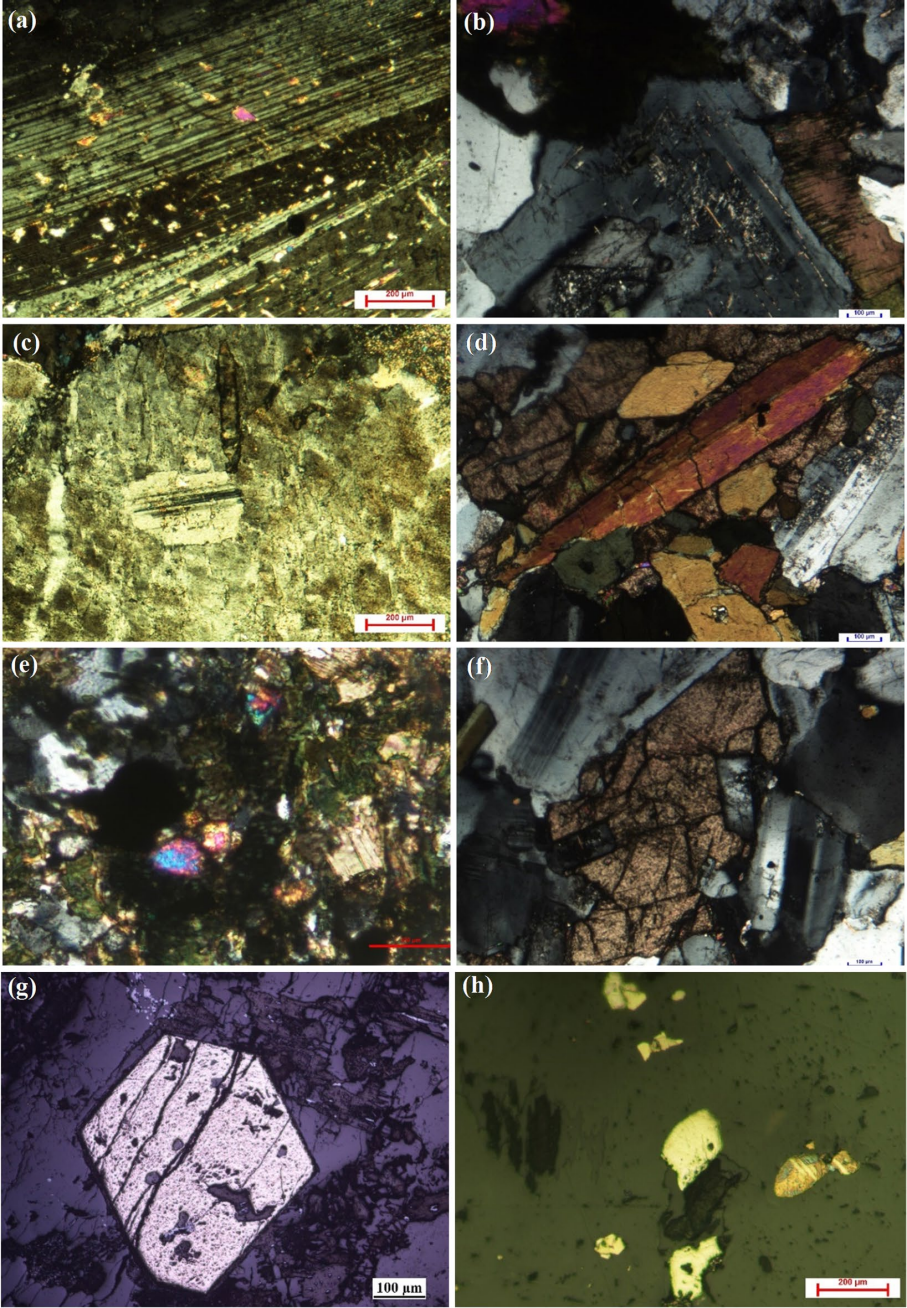
Photomicrographs of the studied granodiorite at Suwayqat El-Arsha, Central Eastern of Egypt. (a) Plagioclase slightly altered to sericite, (C.N.), (b) Zoned plagiocalse associated with altered biotite crystal (chlorite), (C.N.), (c) Orthocalse shows patchy intergrowth, and poikilitically encloses plagioclase, (C.N.), (d) Prismatic hornblende crystal is highly corroded, and associated with rhombic titanite, (C.N.), (e) Zircon enclosed in altered mafic mineral (biotite), (C.N.), (f) Titanite crystal shows rhombic or wedge-shape, (C.N.), (g) Euhedral equant magnetite crystal, reflected light, (h) Tiny pyrite crystals scattered in granodiorite, reflected light.

In hand specimen, granodiorite is coarse-grained, gray to whitish-gray, and spotted with dark mafic grains. Under the microscope, granodiorite is defined strictly by its modal mineralogy specifically the ratio of plagioclase to alkali feldspar and quartz content (Q-A-P)^50^ as mentioned in (Table 3; Fig. 4). It exhibits hypidiomorphic equigranular texture, and is mainly composed of plagioclase, quartz, K-feldspars, biotite, hornblende and opaques (Fig. 5a–h). Titanite, zircon and apatite are accessory minerals, while chlorite, sericite, epidote and iron oxides are alteration products. Plagioclase occurs as subhedral to euhedral prismatic crystals, showing pericline and albitic twinning. It shows normal zoning, and partially to completely altered to sericite, particularly in the core (Fig. 5a, b). Quartz occurs as anhedral to subhedral, medium-grained, and occur as interstitial space fillings between the other essential constituents. K-feldspar occurs as subhedral to anhedral crystals of orthoclase microperthite and showing patchy and flame-type intergrowth (Fig. 5c). Biotite occurs as platy crystals or euhedral to subhedral flakes and corroded by quartz. It is highly alter to chlorite (Fig. 5b), and enclose zircon and apatite. Hornblende manifests as subhedral prismatic crystals, display simple twinning, strong pleochroism and highly altered to chlorite and iron oxides (Fig. 5d). Zircon occurs as prismatic crystals, and poikilitically enclosed in mafic minerals (Fig. 5e). Titanite manifests as euhedral to subhedral rhombic crystals (Fig. 5f). Apatite is found as euhedral prismatic crystals, and predominated enclosed in biotite, hornblende and plagioclase. Opaques are well represented by magnetite and ilmenite with minor pyrite (Fig. 5g, h). Magnetite manifests as cubic and prismatic crystals (Fig. 5g). Ilmenite is found as discrete prismatic crystals without exsolution. Pyrite representes as very tiny cubic crystal that scattered in the rock (Fig. 5h).

**Table 3:** Modal analysis of the studied granodiorite at Suwayqat El-Arsha area.

Samples	Quartz	Alkali feldspar	Plagioclase
Gd29	56	8	36
Gd32	54	12	34
Gd35	52	8	39
Gd38	48	13	38
Gd41	43	12	46
Gd44	42	17	42
Gd47	38	15	47
Gd50	34	16	50
Gd52	30	15	55
Gd55	29	18	53
Gd59	26	17	57
Gd63	27	13	60

### Physico-mechanical properties

**Fig. 6:**
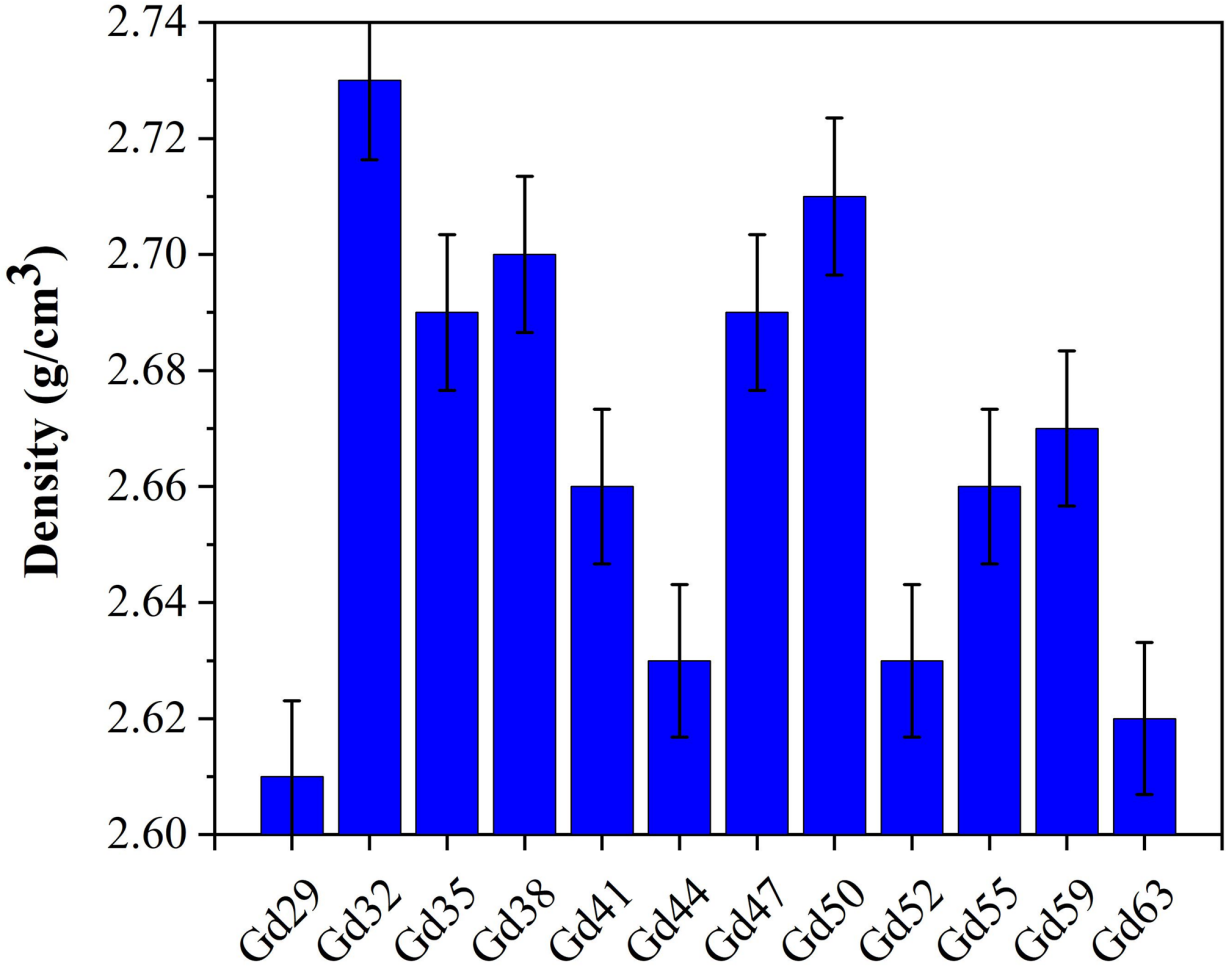
The variation of the density (g/cm^3^) with the grandiorite samples.

The recorded density ranges of the granodiorite samples studied (Table 1) showed some variations from 2.61 to 2.73 g/cm^3^ for this initial sampling demonstrated a slight variation in the analyzed samples. Densities vary in concert with the bulk chemical composition of the rocks discussed in Fig. 6. The highest density recorded was in sample Gd32 (2.73 g/cm^3^) to the lowest density Gd29 (2.61 g/cm^3^). Even though variations exist, the range of densities found in this study is within what would be expected from granodiorite density which typically ranges from about 2.60 to 2.75 g/cm^3^, lending confidence to the measurement and compactness of the sampled granodiorite materials used. Variations in density between the samples is directly related to oxide composition including the ratio of heavy to light oxides. The samples with high contents of Fe_2_O_3_, TiO_2_, CaO, and MgO (Gd29, Gd44, Gd63) have slightly higher density because they contain heavier mineral components such as biotite, amphibole and ilmenite, whereas the samples that are high in SiO_2_ and alkalis (Na_2_O, K_2_O) (Gd32, Gd50, Gd38) have slightly lower densities because they are more felsic and silica-rich, containing a higher percentage of quartz and feldspar mineral than the ferromagnesian mineral phases, both of which contribute to lower bulk density. An important observation is the inverse relationship between SiO₂ content and density, which is a common geochemical trend in granitic rocks, whereby increasing SiO_2_ content leads to lighter, more polymerized mineral structures. Moreover, the low values of loss on ignition (LOI) (< 1 wt%) observed in the samples confirm the limited presence of volatile components, and reinforce their dense, low-porosity texture, which also explains the constants in density values. Comparison of the Fe_2_O_3_, CaO, and MgO (1.99–3.32 wt%, 2.34–3.87 wt%, and 0.88–1.56 wt%, respectively) indicates a small but meaningful compositional influence on density, perhaps related to localized mineralogical differences between the plagioclase-rich and biotite-rich regions of the rock. The relationships between silica enrichment and ferromagnesian oxide concentration provide further explanation for minute density changes of the analyzed samples. Specifically denser samples, particularly Gd32, Gd38, and Gd50, will be associated with an apparent increase in mechanical strength as well as improved radiation shielding capability. Lighter more silica-rich samples will be associated with increased resistance to weathering. Overall, results reinforce the idea that Correspondence to the above results composition impacts the physical behaviours in a minor but effective way, and thus, these granodiorites have adequate well-consolidated textures and density for both construction and radiation shielding applications.

The granodiorite samples (GD29, GD32, and GD35) are selected to study the water absorption rate and exhibited in (Table 2). The absorption values ranged from 0.21% to 0.51% suggesting all tested granodiorite samples have low water absorption characteristics. The lowest absorption value belonged to the GD32 sample (0.21%) which indicates a more compact, and less porous sample likely related to pigment concentration and mineral crystallinity. Oppositely, GD29 showed the highest absorption value (0.51%) suggesting that the sample is slightly more porous or contains a few additional micro-fractures in the sample texture. The GD35 sample absorption was intermediately (0.38%) reflective of moderate compactness. In general, these tested low water absorption aggregates support the conclusion that the granodiorite samples possess low permeability and therefore excellent water resistance and structural integrity, which is crucial for construction and radiation shielding applications. Low permeability is a requirement for extended use and stability against environmental variability. The differences in absorption behavior may be attributed to mineralogical differences between granodiorite samples, grain size distribution and degree of alteration.

The compressive strength of the granodiorite samples, as illustrated in the Table 2, is significantly variable with values ranging from 953 to 1146 kg/cm^2^. The sample with the greatest compressive strength in this study was Gd32 (1146 kg/cm^2^), which likely indicates that this sample has a high degree of mineralong > interlocking, compact texture, and low porosity that results in the high mechanical performance of the sample. This feature likely occurs due to high contents of silica and alumina which enhance the stability of durable silicates within the rock matrix. The sample Gd41 had the lowest compressive strength (953 kg/cm^2^) which may be the result of slight imperfections of texture or more significant alteration of porphyroblasts of minerals such as biotite and amphibole. Other samples included in the study (Gd29, Gd35, and Gd38) demonstrated intermediate strength with values ranging from 1016 to 1102 kg/cm^2^ indicating good structural integrity and relative low variability when mineral composition was analyzed. In general, the results indicated that all granodiorite samples have high compressive strength and could be classified as mechanically strong and durable materials with structural and shielding properties. Variation among samples is likely related to mineralogical composition, distribution and size of grains, and crystallization texture which are all important to mechanical performance and preparation for construction and radiation protection applications.

**Fig. 7:**
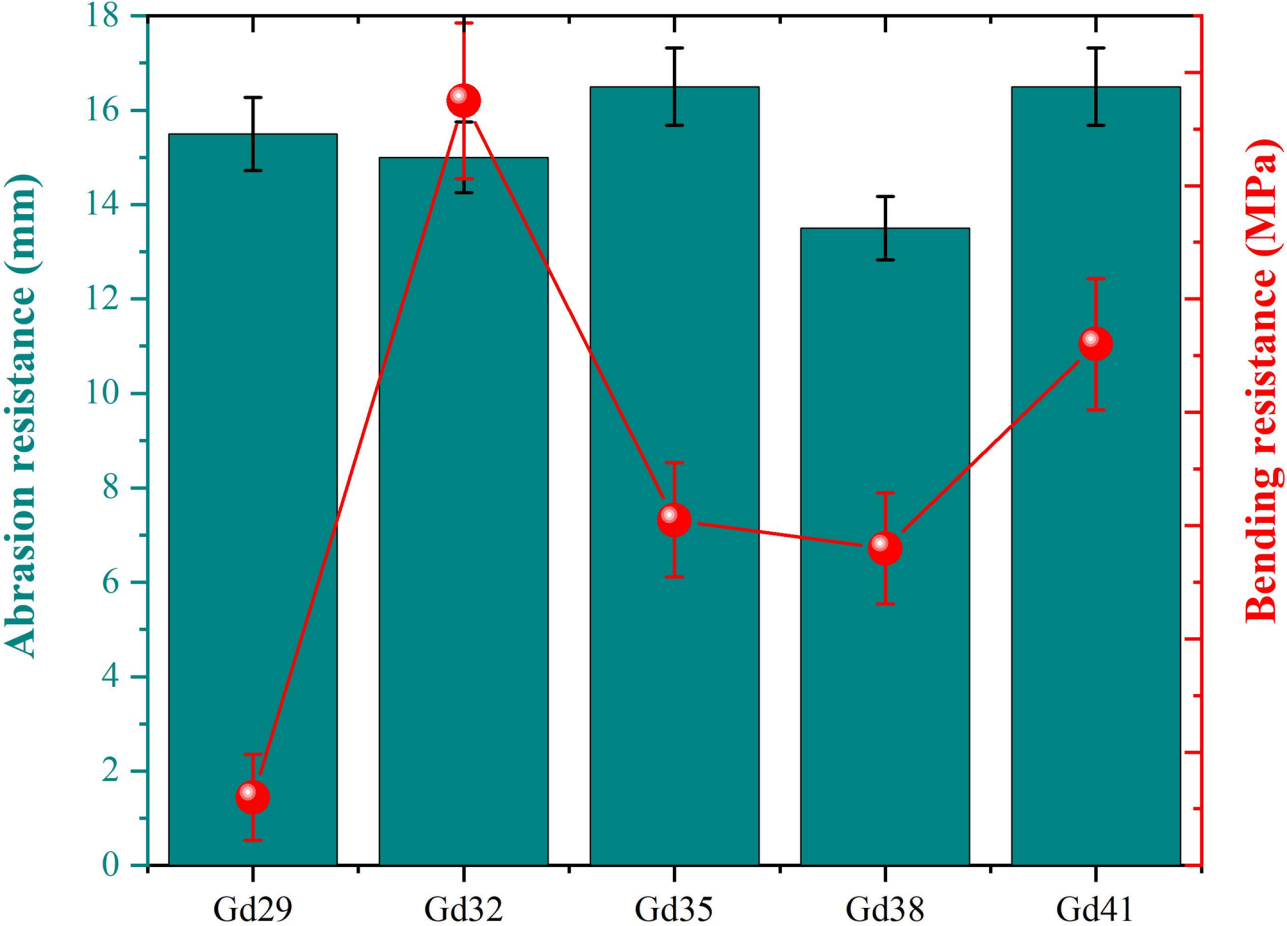
Variation of abrasion resistance (mm) and bending (flexural) resistance (MPa) for the investigated granodiorite samples.

The abrasion resistance testing results for the granodiorite specimens are provided in the following Fig. 7, indicating that abrasion loss values range from 13.5 to 16.5 mm, showing good to very good resistance to wearing of the surface. Among the specimens examined, Gd38 showed the lowest abrasion value (13.5 mm) representing the highest resistance to abrasion and surface durability. The increased resistance to the Gd38 specimen can be attributed to its compact crystalline texture and high interlocking of mineral grains, especially for quartz and feldspar, which increases the hardness and resistance to wear. In comparison, Gd35 and Gd41 displayed the highest abrasion values (16.5 mm), which suggests relatively lower resistance to wear, possibly related to these specimens’ higher quantity of softer minerals such as biotite or possibly some minor micro-fractures in the sample that reduce surface cohesion. Each of the other samples (Gd29, Gd32, and Gd44) showed values between the previous two values showing intermediate results from 15.0 to 15.5 mm, which were representative of average geological, mechanical strength, and mineralogical composition. Overall, the results obtained suggest that the granodiorite samples show good abrasion resistance suitable for heavy-use construction applications like flooring, pavements, or cladding; where cementitious structure calls for durability over long-term mechanical use. The small differences in abrasiveness can be attributed to the variation in grain size, mineralogy, and the degree of cementation, all of which affect the mechanical wearing behavior of the rock.

Based on the data shown in Fig. 7, the flexural (bending) strength results for the granodiorite samples displayed ranges of 15.2 to 27.5 MPa, signifying considerable differences in mechanical performance among the samples. The Gd32 sample demonstrated the greatest amount of bending resistance (27.5 MPa) and therefore greater ability to resist deformation upon stress applications. The ability of the Gd32 sample to perform in this manner was likely due mainly to the high density and compact crystalline structure and overall balanced mineral composition of the sample, particularly with a greater amount of silica and feldspar minerals, which are known to have rigidity and intergranular bond strength in rocks. In contrast, Gd29 showed the lowest bending strength (15.2 MPa) and the texture heterogeneity or micro-crack features and the relatively soft minerals, biotite, are likely to justify its inferior integrity. Other samples, Gd35 (20.1 MPa), Gd38 (19.6 MPa), and Gd41 (23.2 MPa), had intermediate values that displayed consistent results suggesting likely applicability for most structural applications. In general, the findings show that granodiorite has good flexural strength and is therefore a suitable natural material for the construction, flooring, and radiation shielding industries. The differences in bending resistance are attributed to mineralogical composition, grain size distribution, and crystallinity of the granodiorite itself. These three factors ultimately dictate the rock’s resistance to external force and overall mechanical durability.

After comparison with international performance standards, it was revealed that Suwayqat El-Arsha Granodiorite has mechanical properties well above the minimum required thresholds for both dimension and structural stones. Using the ASTM C170/C170M-17 standards to define compressive strength of dimension stones^42^, granite needs to have a compressive strength greater than approximately 1000 kg/cm^2^, and most of our tested granite samples recorded values between 1016 and 1146 kg/cm^2^, indicating that they all met or exceeded this benchmark. Water absorption values of Suwayqat El-Arsha Granodiorite were recorded from 0.21% to 0.51%, which is significantly lower than the upper limits specified in the absorption and bulk specific gravity standards of the ASTM C97/C97M-18^51^. Most granites of good quality have an absorption value of less than 0.4 to 0.7%. The abrasion resistance of Suwayqat El-Arsha Granodiorite, using values of 13.5 to 16.5 mm, was evaluated using ASTM C241/C241M-20, as well as EN 14157:2004^44,52^, and are considered to be equivalent or superior to commercial-grade granite used for flooring and paving. From these evaluations, it is clear that Suwayqat El-Arsha Granodiorite meets or exceeds the International Performance Standards for durability and structural applications.

### Shielding capability

**Fig. 8:**
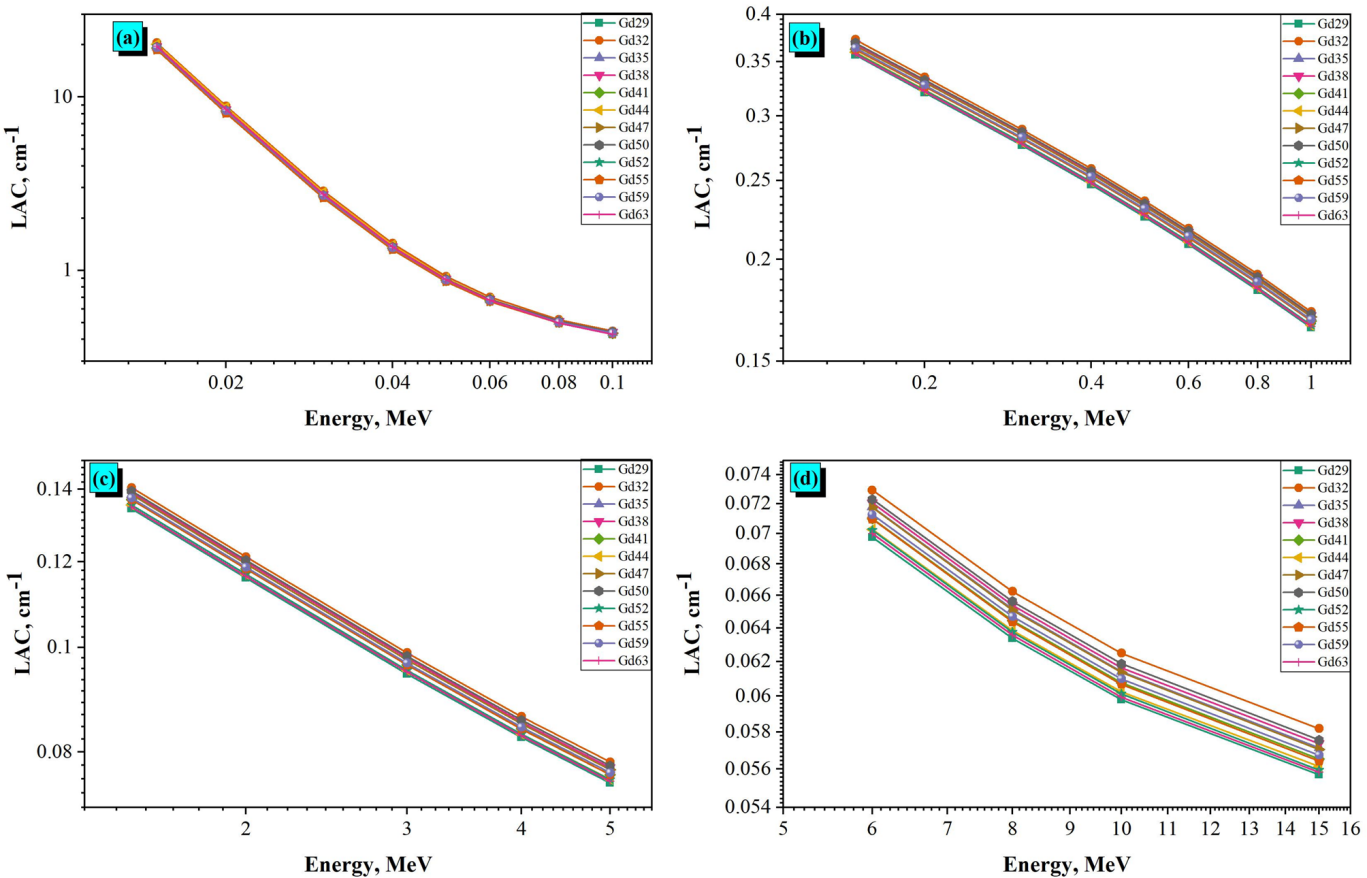
The linear attenuation coefficient (LAC) of grandiorite samples at various E_γ_ values, (a) 0.015-0.1 MeV, (b) 0.1-1 MeV, (c) 1-5 MeV, (d) 5-15 MeV.

The LAC, cm^− 1^ were calculated for twelve granodiorites (Gd29 - Gd63) at photon energies 0.015–15 MeV. The results (Fig. 8a–d) reveal a strong dependence on photon energy and a lesser dependence on sample chemistry and density. As expected, the LAC decreases exponentially with photon energy for the same reason: the photon interaction mechanisms begin to transition. This energy range can be divided into four different regions, each of which has a dominant attenuation process and an influence from the composition and chemistry of the rocks.

Figure 8a shows that the LAC values are at their maximums at low photon energies (about 20.51 cm^− 1^ for Gd32 at 0.015 MeV; down to 0.43 cm^− 1^ for Gd32 at 0.1 MeV). The photoelectric absorption process dominates at low energies and is a function of both the photon energy (E^− 3^ power) and the effective atomic number (Z_eff_) of the material^53,54^. Granodiorite samples enriched with heavier oxides (Fe_2_O_3_, TiO_2_, and CaO) show relatively higher LAC values in the low photon energy range—for example, Gd29 (Fe_2_O_3_ = 3.32 wt%, TiO_2_ = 0.77 wt%) and Gd44 (Fe_2_O_3_ = 3.26 wt%, TiO_2_ = 0.74 wt%). Alternatively, a sample with high silica (SiO_2_ > 71 wt%) contents (Gd47, Gd50, Gd55) shows lower low energy LAC coefficients since they are comprised of light material and have a lower mean atomic number. These relationships confirm that at low photon energies, the photoelectric effect is the dominant process and not unexpectedly, the higher Z_eff_ values due to the enrichment and the lighter atomic number reduction biased the attenuation ability of the aforementioned compositions. In the intermediate energy range (Fig. 8b), the LAC values decrease rapidly from approximately 0.43 cm^− 1^ at 100 keV to approximately 0.17 cm^− 1^ at 1 MeV; this decrease reflects the dominance of Compton scattering, which depends primarily on electron density in the material rather than the atomic number of the underlying element. The other samples showed only minor LAC differences ( < ± 0.01 cm^− 1^) that are probably attributable to small differences in composition among the granodiorite samples. Even though all samples are compositionally similar, the LAC values for samples with slightly higher Fe_2_O_3_ and CaO, such as Gd29 and Gd44, tend to be higher throughout this region. The mean attenuation coefficient for all samples at 0.5 MeV is approximately 0.23 cm^− 1^, which is consistent with similar or expected values recorded for granitic rocks^55,56^. More silicic samples (Gd47, Gd55), with lighter compositions have lower LAC values (~ 0.226 cm^− 1^) which again, is consistent with lower electron density. The relative stability of LAC across all samples in this energy range indicates that the granodiorites studied, with SiO_2_ values ranging from 68.2 to 71.7 wt%, and average Al₂O₃ of ~ 14.7 wt%, were similar in composition.

With increased photon energies (Fig. 8c), the LACs continue their decline more steadily, moving from ~ 0.165 cm^− 1^ at 1 MeV to ~ 0.075 cm^− 1^ at 5 MeV. For this range, Compton scattering remains the main interaction process, with pair production (above 1.02 MeV) growing in contribution. The slower decline compared to lower energies suggests the interaction probability is now less dependent on the photon energy. Changes in LAC among the samples is again subtle; however, Gd32 and Gd35 have somewhat greater attenuation values in this range, presumably due to their moderate concentrations of Fe_2_O_3_ and K_2_O. In contrast, samples comparable to Gd47 (Fe_2_O_3_ = 1.99 wt%) and Gd50 (2.31 wt%) exhibit lower LAC, reflecting a lower Z_eff_ and density. The dominance of composition in this range corroborates the role of iron- and alkali-bearing oxides in increasing gamma-ray interaction probability at moderate-to-high energies. In the very-high-gamma energy (Fig. 8d), the LAC values decrease only moderately from ~ 0.075 cm^− 1^ at 5 MeV to ~ 0.055 cm^− 1^ at 15 MeV. The LAC values of different samples converge at higher energies, but samples differ by a certain amount, even if the differences are small. For example: At 5 MeV the LAC of Gd29 was 0.0749 cm^− 1^ and that of Gd32 was 0.0783 cm^− 1^. The LAC values of two additional samples (Gd63 and Gd44) at 10 MeV were narrowly spaced between 0.0599 cm^− 1^ and 0.0602 cm^− 1^, respectively. The same holds true at 15 MeV where the LAC of the samples ranged from 0.0556 to 0.0581 cm^− 1^, which means that even though all sample’s curves appear similar, as illustrated in Fig. 8, they demonstrate a small consistent influence of high-Z minerals and density throughout the range of energies utilized in the study.

At these energies, pair production is the main event, and attenuation changes are less compositionally-dependent. The nearly constant attenuation response in all granodiorites suggests the probability of interaction between photons and matter is nearly energy-independent. The limited variation in LAC (i.e. < 0.003 cm^− 1^) values between samples reinforces the overall geochemical consistency in granodiorites. In select samples with slightly larger Fe_2_O_3_, TiO_2_, and CaO concentrations (e.g. Gd29 and Gd44) provide marginally better attenuation performance at the high energies, suggesting that these oxides provide some small contribution to the increase of Z_eff_.

**Fig. 9:**
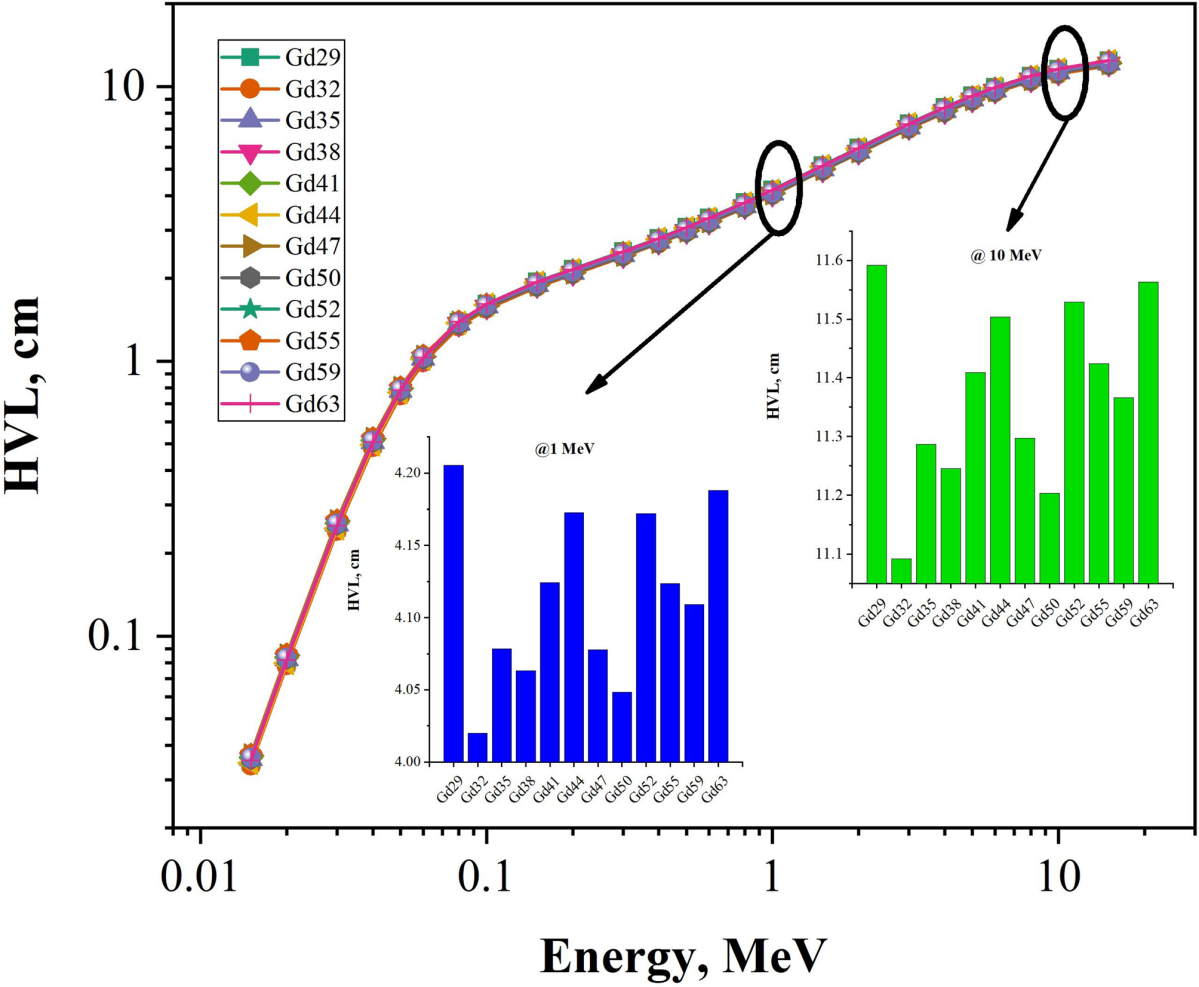
The half-value layer (HVL) as a function of photon energy E_γ_ values 0.015-15 MeV are presented for the granodiorite samples (Gd29 – Gd63). The two inset panels (1 MeV and 10 MeV) below the HVL versus photon energy plot help the reader to more clearly compare HVLs for the samples along the selected photon energy scale.

**Fig. 10:**
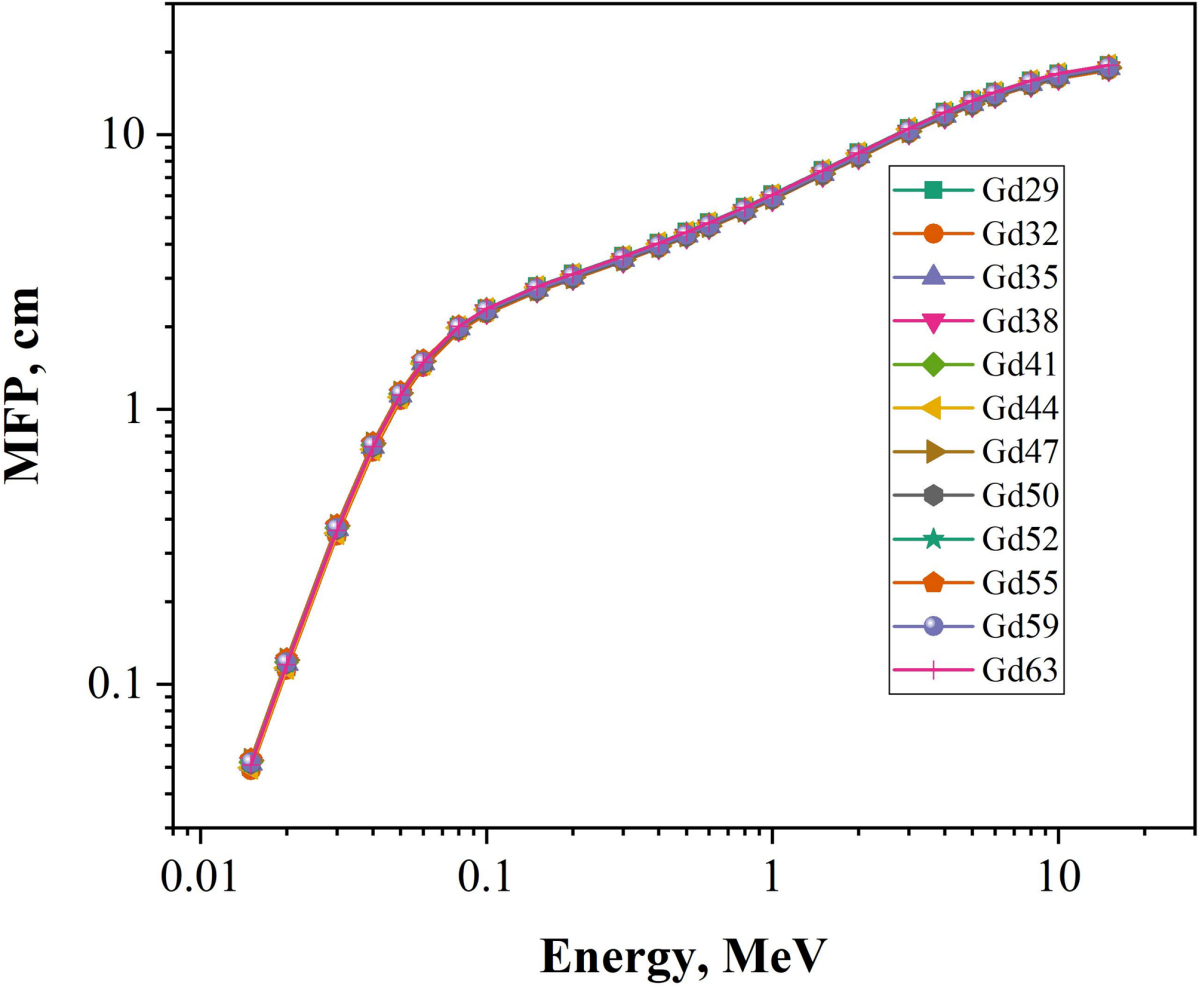
The mean free path (MFP, cm) of grandiorite samples at various E_γ_ values 0.015-15 MeV.

The results for HVL and MFP in Figs. 9 and 10 have a clear and consistent relationship with respect to the granodiorite samples when analysed based on photon energy (Gd29-Gd63). There is also a strong increase in both parameters as the photon energy increases. This relationship between photon energy and HVL and MFP reflects the change in probability of a gamma ray interacting with matter. As the energy increases from the photoelectric-dominant energy levels (0.015 MeV to 0.05 MeV) to the intermediate and higher levels for Compton Scattering and Pair Production (up to 15 MeV), the probability of interaction decreases. The HVL and MFP for both granodiorite samples at low energies (0.034 to 0.086 cm for HVL and 0.048 to 1.14 cm for MFP respectively) demonstrate that these granodiorites have excellent attenuation properties; while at 15 MeV, both HVL and MFP reach their maximum values (HVL = 11.9–12.4 cm and MFP = up to 17.9 cm). In fact, in general, while the samples appear to be similar, minor compositional differences in density and in the abundance of heavier-Z elements such as Fe, Ca, and Ti provide small, but significant differences in the HVL and MFP. Gd32 and Gd44, the densest samples in the test, always exhibit lower HVL and MFP values than the other three samples, with values 5.80–6.07 cm (1 MeV MFP) and 17.19–17.95 cm (15 MeV MFP) respectively. These lower HVL and MFP values support the findings that Gd32 and Gd44 have higher effective atomic numbers and electron density compared to other granodiorite samples; therefore, providing more effective attenuation of photons and increasing the probability of photon interaction in these samples. Thus, the HVL-MFP results illustrate that granodiorite containing heavy-element enrichment has the greatest attenuating capacity to provide effective gamma-ray shielding and, therefore, can be utilised as a natural option for gamma-ray shielding applications.

**Fig. 11:**
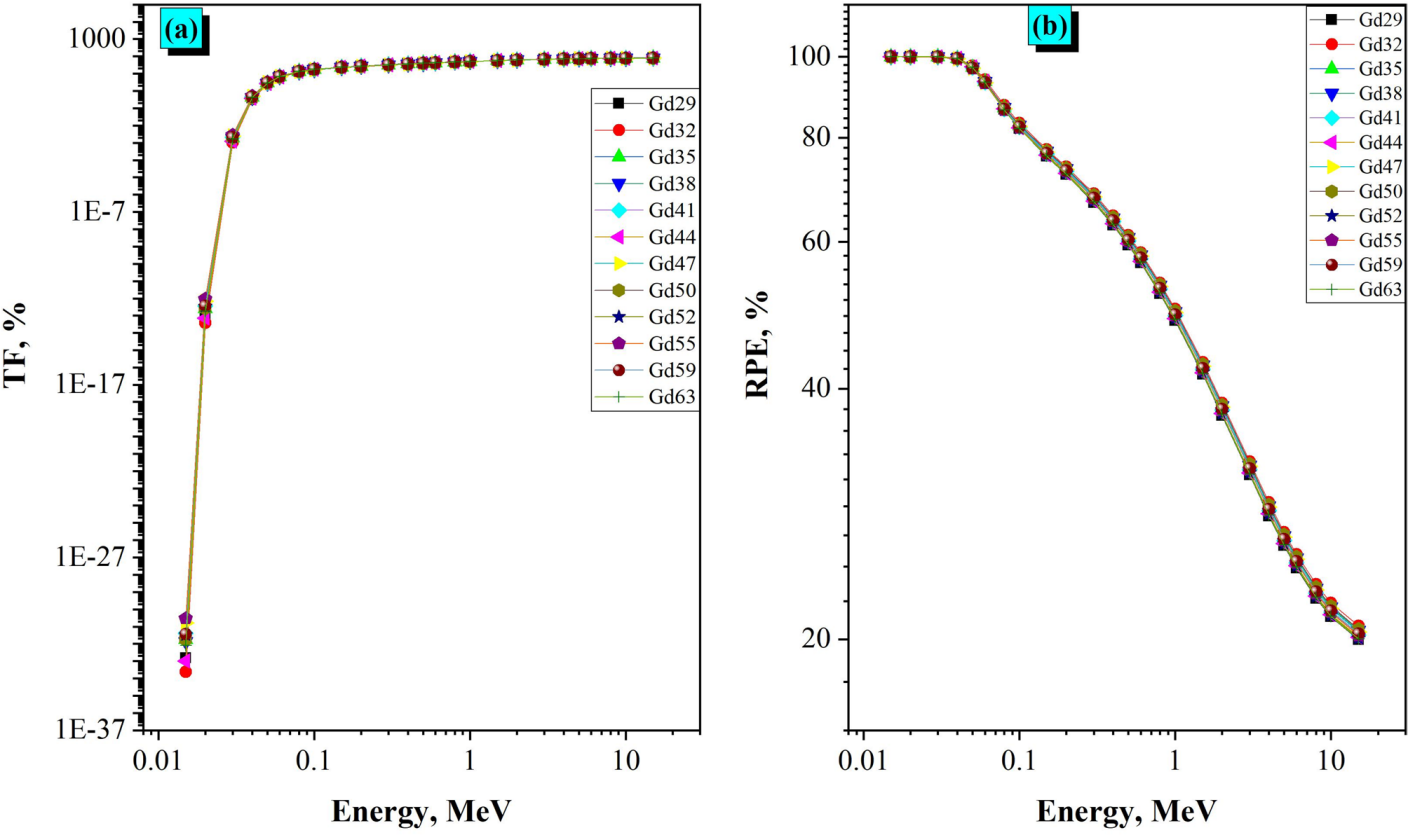
(a) The transmission factor (TF, %) and the radiation protection efficiency (RPE, %) of grandiorite samples at various E_γ_ values 0.015-15 MeV.

The transmission factor (TF %) serves as a paramount gauge of the shielding capability of the granodiorite samples in terms of gamma radiation. The relationship of TF with photon energy for a sample thickness of 4 cm is shown in Fig. 11a for gamma photon energies ranging from 0.015 to 15 MeV. The results have shown that the TF values subsequently, increased with increasing photon energy, which is expected from the basic principles of gamma-ray interactions. At very low energy (0.015–0.02 MeV) the TF values were close to zero for all granodiorite samples, suggesting full photon absorption as a result of the photoelectric effect being strong. Beyond the energy of 0.03 MeV, the TF began to rise slowly with some intermediate photon energies (0.4–0.6 MeV), reaching values around 40–43% where Compton scattering was the dominant mechanism. As photon energy increases (greater than 1 MeV), the TF values continued to increase, but at a slower rate and then tended to stabilize within the range of 50–80% in the 1–15 MeV range. This behavior demonstrates a lower probability of photon interaction occurring within the material as photon energy increases. Of all the samples tested, Gd29 had the lowest TF values, demonstrating superior attenuation ability, whereas Gd63 exhibited the highest TF and hence somewhat lower shielding ability. The differences in TF values can be attributed to small differences in composition ranging between the granodiorite samples, specifically in heavy-element content, which alters the probability of photon interaction. In general, the results indicate that granodiorite has strong low- and medium-energy gamma photon shielding properties as a natural radiation shielding material.

The variation in radiation protection efficiency (RPE %) of the granodiorite samples as a function of photon energy is insightful regarding their shielding performance against gamma radiation. The RPE values presented in this study (Fig. 11b) decrease progressively as photon energy increases, due to lower probabilities of photon–matter interactions as photon energy increases. At the lower photon energy range of 0.015–0.03 MeV, all samples displayed an RPE of 100%, indicating complete attenuation of low energy photons, consistent with the greater role of the photoelectric absorption process. As they increased in energy to about 0.05–0.4 MeV, the RPE steadily decreased down to values about 63–97%, indicating that Compton scattering was starting to have a more significant role in attenuation as the primary interaction process. After 1 MeV, a more drastic decline in RPE occurred, ranging from about 48–50% at 1 MeV to 20–21% at 15 MeV, suggesting a markedly diminished attenuation capacity; this was due to photons being more focused on material penetration than interaction at elevated energies. Gd32 consistently demonstrated the highest RPE values compared to other samples at almost all photon energies, signifying stronger shielding capability due to the higher density and the availability of bigger atomic weight elements and shielding capability. In contrast, Gd63 always had the lowest efficiency. Overall, these results provide evidence that granodiorite samples would be effective radiation shielding materials at low and intermediate energies, namely Gd32, while still being considered eco-friendly, stable lasting materials, and low cost for when applied in radiation shielding and nuclear safety features. All shielding results from this study have been estimated using Phy-X/PSD; however, while these estimates may provide a reliable estimate, the results obtained using this method do not account for real-life influences on attenuation, e.g., micro-heterogeneity; effects of grain boundaries; surface conditions; and geometry of the radiation beam will affect actual attenuation. Therefore, before using these estimates to explain gamma attenuation characteristics, it is necessary to validate and verify through experimental measurements of gamma attenuation.

The current studies MAC values for the granodiorite samples were compared against those of natural and synthetic materials by a few different authors (Table 4) at a photon energy of 0.662 MeV. The granodiorite samples yielded MAC values that, for the most part, were grouped closely together in a narrow range of 0.07994–0.07998 cm^2^/g, which indicates a high degree of consistency and homogeneity in composition. These MAC values were very similar to those of silicate-based rocks, providing added confidence supporting the application of granodiorite as a breeding material for shielding from radiation exposure. As noted in Table 4, comparison with prior studies show the MAC values obtained in this study were presented in great agreement with that of natural granite with corresponding MAC values of 0.0802 cm^2^/g^55^, and the synthetic granite (0.0798 cm^2^/g) quantified by^57^ for experiments with only slight variations under stated experimental uncertainty. Such close similarities are primarily based upon similarities in elemental compositions that contain moderate- to high-Z elements such as Fe, Ca, or K which are responsible for increasing interaction probabilities compared with photoelectric and Compton scattering. In contrast, the current study found that the MAC values of Fe-rich silicate granodiorites were marginally higher than the MAC of limestone (0.065cm^2^/g) reported by^11^. This was not surprising, as average atomic numbers and densities of carbonate minerals were lower than those of granodiorite, leading to decreased MAC for limestone. The granodiorites values were also slightly lower than that of basalt with higher zero values of Fe and Mg oxides generating an overall greater effective atomic number. Both granodiorites were reported with near-MAC values of close to that of marble (0.0784 cm^2^/g) and jet black granite (0.0806 cm^2^/g). All of these reports support the assumption that granodiorite is in the expected attenuation range of dense silicate rock. In conclusion, the close correspondence of the present results with published results demonstrates that granodiorite has similar gamma-ray attenuation properties as traditional shielding materials like granite and basalt while providing benefits of natural abundance, mechanical durability, and attractiveness. As a result, the granodiorite that was studied could be recommended as a cost-effective and environmentally friendly alternative for structural and radiation shielding purposes in laboratories, nuclear establishments and materials subject to ionizing radiation.

**Table 4:** Comparison of mass Attenuation coefficient (MAC, cm^2^/g) of the studied granodiorite samples with previous studies.

Sample	MAC (cm^2^/g)	Reference
Gd29	0.07994	Present study
Gd32	0.07995	Present study
Gd35	0.07996	Present study
Gd38	0.07996	Present study
Gd41	0.07997	Present study
Gd44	0.07994	Present study
Gd47–Gd63 (range)	0.07994–0.07998	Present study
Granite	0.0802	
Basalt	0.081	
Limestone	0.0765	
Marble	0.0784	
Jet Black Granite	0.0806	
Synthetic Granite (MCNPX)	0.0798	

## Conclusion

Granodiorite has good mechanical performance and good potential for shielding against radiation, allowing for application as an alternative stone for structural and nuclear applications. Petrographic and geochemical results indicate that the granodiorite consisted primarily of quartz, plagioclase, K-feldspar, and accessory biotite and hornblende, being silica-rich compositions (SiO_2_ = 68.2–71.7 wt %). The physical properties show moderate density (2.61–2.73 g/cm^3^) and low water absorption (0.21–0.51%), indicating the high-density strength and a durable texture for its purposes. The mechanical tests demonstrated the rock displays high compressive strength (953–1146 kg/cm^2^), flexural strength (15.2–27.5 MPa), and abrasion resistance (13.5–16.5 mm), demonstrating mechanical strength and durability. The radiation attenuation remained LAC values decreased from 20.51 cm^to 1^ at 0.015 MeV to 0.055 cm^-1^ at 15 MeV, while HVL values increased from 0.034 cm to 12.4 cm. The low MFP (0.048–17.9 cm) and high RPE (up to 100% at ≤ 0.03 MeV) indicated efficient gamma-ray attenuation, particularly for denser samples that contain Fe and Ti elements like Gd32 and Gd44. The findings of this investigation indicate that Suwayqat El-Arsha granodiorite is capable of achieving an intermediate to high mechanical performance and promising results for its ability to serve as a gamma-ray shielding material based on computational calculations. Given these preliminary results, caution is warranted when considering their potential applicability as a construction and shielding material. Among study limitations are reliance on computed models rather than direct experimental verification, as well as the small size of the sample represented within the lithological unit studied. These findings concluded that granodiorite is an environmentally conscientious natural material due to its structural and low permeability, as well as having strong radiation protection efficiency, and can be utilized for green shielding and sustainable construction practices in nuclear and industrial settings.

## Data Availability

The datasets used and/or analysed during the current study available from the corresponding author on reasonable request.
